# DArTseq-Based, High-Throughput Identification of Novel Molecular Markers for the Detection of Fusarium Resistance in Maize

**DOI:** 10.3390/ijms262110534

**Published:** 2025-10-29

**Authors:** Maciej Lenort, Agnieszka Tomkowiak, Aleksandra Sobiech, Jan Bocianowski, Karolina Jarzyniak, Przemysław Olejnik, Tomasz Jamruszka, Przemysław Gawrysiak

**Affiliations:** 1 Department of Genetics and Plant Breeding, Poznań University of Life Sciences, Dojazd 11, 60-632 Poznań, Poland; lenort.maciej@up.poznan.pl (M.L.);; 2 Plant Breeding and Acclimatization Institute—National Research Institute in Radzików, 05-870 Błonie, Poland; 3 Department of Mathematical and Statistical Methods, Poznań University of Life Sciences, Wojska Polskiego 28, 60-637 Poznań, Poland; jan.bocianowski@up.poznan.pl; 4 Department of Biochemistry and Biotechnology, Poznań University of Life Sciences, Dojazd 11, 60-632 Poznań, Poland; 5 Institute of Environmental Biology, Faculty of Biology, Adam Mickiewicz University in Poznań, Uniwersytetu Poznańskiego 6, 61-614 Poznań, Poland

**Keywords:** DArTseq, NGS, GWAS, *Fusarium*, resistance, Maize

## Abstract

Modern maize breeding worldwide relies on a broad range of molecular genetics research techniques. These technologies allow us to identify genomic regions associated with various phenotypic traits, including resistance to fungi of the genus *Fusarium*. Therefore, the aim of this publication was to identify new molecular markers linked to candidate genes that confer maize resistance to *Fusarium* fungi, using next-generation sequencing, association mapping, and physical mapping. In the study, a total of 5714 significant molecular markers related to maize plant resistance to *Fusarium* fungi were identified. Of these, 10 markers were selected that were significantly associated (with the highest LOD values) with the disease. These markers were identified on chromosomes 5, 6, 7, 8, and 9. The authors were particularly interested in two markers: SNP 4583014 and SilicoDArT 4579116. The SNP marker is located on chromosome 5, in exon 8 of the gene encoding *alpha-mannosidase I MNS5*. The SilicoDArT marker is located 240 bp from the gene for peroxisomal carrier protein on chromosome 8. Our own research and the presented literature review indicate that both these genes may be involved in biochemical reactions triggered by the stress caused by plant infection with *Fusarium* fungal spores. Molecular analyses indicated their role in resistance processes, as resistant varieties responded with an increase in the expression level of these genes at various time points after plant inoculation with *Fusarium* fungal spores. In the negative control, which was susceptible to *Fusarium*, no significant fluctuations in the expression levels of either gene were observed. Analyses concerning the identification of *Fusarium* fungi showed that the most abundant fungi on the infected maize kernels were *Fusarium poae* and *Fusarium culmorum.* Individual samples were very sparsely colonized by *Fusarium* or not at all. By using various molecular technologies, we identified genomic regions associated with maize resistance to *Fusarium* fungi, which is of fundamental importance for understanding these regions and potentially manipulating them.

## 1. Introduction

Currently, maize for grain is grown on approximately 197 million hectares of land worldwide, making it the second most economically important crop after wheat. For comparison, the areas under wheat cultivation and rice cultivation are 216 million hectares and 165 million hectares, respectively [1]. The annual global production of maize grain is currently 1137 million tons, significantly higher than the production of rice and wheat. Over the last quarter-century, maize production has more than doubled, a trend favored by both a significant increase in yields and an expansion into ever-wider areas [1]. Among the three cereals mentioned, maize yields have increased by almost 2 tons in 25 years (from 3.9 to 5.8 t/ha). The aforementioned intense increase in maize yields would not have been possible without biological progress. This progress can be described as an ecological approach to intensifying agricultural production, based on the genetic improvement of plants that become more efficient in using natural forces and industrial means of production [2]. These plants are also of better quality from the point of view of human requirements, which consequently leads to a reduction in the costs incurred for their production. It is currently assumed that maize breeding priorities include introducing varieties with higher utility value, i.e., greater yield and improved nutritional, feed, and technological value of the resulting yield. It is also important to increase plant resistance to both biotic and abiotic stresses [3].

Climate change poses a serious threat to world food security. The currently prevailing weather conditions are a factor that, to a greater extent, affects the infection of maize kernels by fungi of the genus *Fusarium* [4]. Since regular temperature measurements began, it has been noticed that the climate has become warmer [5]. A temperature increase of 1 °C would lead to a 3.4% decrease in maize yields [6,7]. In addition, epidemics are often facilitated by the emergence of new species of pests and the invasive spread of existing ones into neighboring areas. Based on research conducted in recent years, it is estimated that maize diseases cause annual yield losses of up to 30% [8]. The quality of the grain yield also significantly deteriorates, as early infection of plants by fungi and bacteria causes dwarfing of the grain, a decline in nutritional value, and a reduction in the quality of the resulting feed [9].

In addition to the pressure from pests and a changing climate, the economic potential of maize production areas is also affected by the European corn borer [*Ostrinia nubilalis* (Hübner)], which is a maize pest [10,11]. There are usually two generations of corn borer larvae per year: the first generation attacks plants in the middle or at the end of the vegetative phase, and the second generation in the reproductive phase (from the early milky stage to full maturity) [12]. Furthermore, the second-generation larvae play a very important role in promoting infection by fungi of the genus *Fusarium* and the production of mycotoxins in maize kernels [13]. It is a disease with a complex etiology, and the causative factors include, among others: *Fusarium graminearum*, *Fusarium culmorum*, *Fusarium avenaceum*, *Fusarium verticillioides*, *Fusarium subglutinans*, and *Fusarium proliferatum*. The most common cause of ear rot is the fungi *Fusarium graminearum* (producing deoxynivalenol—DON and zearalenone—ZEA) and *Fusarium verticillioides* (producing fumonisins—FUM) [14]. *Fusarium verticillioides* (Sacc.) Nirenberg, is the most harmful pathogen widespread on all continents, with greater aggressiveness in warmer climate regions [15]. *Gibberella fujikuroi*, which is the perfect (teleomorph) stage of *Fusarium graminearum*, is also described in the literature. Often, the symptoms of *Fusarium* disease, caused by fungi of the genus *Fusarium,* are not clearly visible on the ear, but the infection progresses internally, leading to the accumulation of mycotoxins [16,17]. Following the discovery of toxins produced by fungi of the genus *Fusarium*, the damage caused by this fungus was fully understood. Mycotoxins can cause many diseases in humans, including various types of allergies, hormonal disorders, and cancers (these toxins activate oncogenic cells). Their presence in feed poses a significant threat to the health of animals, particularly pigs and poultry, as it increases their susceptibility to infectious agents. Under standard conditions, these agents would not be able to cause disease without the additional action of metabolites from toxin-producing fungi. Furthermore, they negatively affect production results [14]. Most countries have introduced new regulations concerning the permissible levels of mycotoxins in food and feed. In 2007, the European Union introduced standards specifying the maximum content of mycotoxins in unprocessed maize grain (EC No 1126/2007). If the DON content in unprocessed grain exceeds 1700 μg/kg, ZEA 350 μg/kg, and FUM 4000 μg/kg, such grain is not eligible for use as feed. Furthermore, in the case of maize, the use of fungicides is challenging and often ineffective, as it is difficult to accurately assess the severity of the disease [18].

Modern resistance breeding in maize worldwide is based on a wide range of research techniques in molecular genetics. The introduction of molecular tools into breeding and the rapid progress in next-generation sequencing (NGS) have enabled the sequencing of the genomes of many crop species, including maize. The Illumina method gave rise to the development of Genotyping-by-sequencing (GBS) [19] and DArTseq procedures [20]. In this study, DArTseq technology was used to identify molecular markers linked to genes that confer maize resistance to fungi of the genus *Fusarium*. This technology reduces the complexity of the genome by digesting it with restriction enzymes, sequencing short reads, and then analyzing the results. The choice of a combination of restriction enzymes allows for the isolation of highly informative fragments of the genome with a low copy number. Up to 90% of the obtained DArTseq markers are complementary to unique genomic sequences [21,22]. The extensive genotypic data obtained from NGS can be used for association mapping. Genome-wide association studies (GWAS) have therefore become a powerful methodology for studying genetic variation and identifying the relationship between a trait and the underlying genetic variation by using historical recombination events [23]. Association mapping involves searching for a genotype-phenotype correlation in unrelated individuals using specialized statistical methods [24,25,26]. The association mapping approach provides opportunities for generating good-quality markers for marker-assisted selection (MAS). Functional markers closely linked to a trait reflect gene polymorphisms that directly cause phenotypic variation. Association mapping offers opportunities to identify specific markers across a wide spectrum of genetic resources. The potential of association mapping results from the probability of obtaining higher resolution, thanks to the use of a larger number of recombination events in the history of germplasm development [27]. Thus, association mapping has become a promising approach compared to traditional mapping. There are two main types of association mapping: genome-wide association mapping (GWAM) and candidate gene association mapping (CGAM). The GWAM approach examines genetic variation across the entire genome in order to find association signals for various complex traits, while CGAM correlates DNA polymorphisms in selected candidate genes with the trait of interest [3]. There are many examples of the successful application of association analysis in cereals, mainly in maize. Recently, GWAM has become a powerful tool for analyzing the genetic architecture of complex traits of various crop species [3]. Initially, association mapping performed in maize [28] did not take into account population structure. This error was corrected by Pritchard, who, in 2001, in research on maize, took population structure into account [29]. Genome mapping showed that resistance to *Fusarium* is controlled by many genes with relatively small effects, which differ depending on the environment and population [30]. Although different maize inbred lines and hybrids exhibit varying genetic variation in terms of resistance to fungi of the genus *Fusarium*, there is no evidence of maize genotypes with full resistance to this pathogen [31]. The identification of new resistance genes is very important in order to find a lasting solution to the problems associated with *Fusarium* in maize production. Several studies have identified QTLs related to resistance to fungi of the genus *Fusarium*, which resulted in a reduction in the accumulation of fumonisins, using crossing populations [32,33,34]. Zila et al., 2013 [35], 2014 [36] conducted GWAS tests on maize to detect an SNP associated with increased resistance to *Fusarium*. They identified ten SNP markers significantly associated with resistance to this pathogen. The authors identified SNP markers related to the defense response in five genes or in their vicinity, which had not been previously correlated with disease resistance, but whose predicted gene functions included the programmed cell death pathway. They identified QTL regions related to fusarium resistance on chromosomes 1, 4, 5, and 9. Similarly, De Jong et al., 2018 [37], through GWAS analyses, identified regions related to *Fusarium* resistance on chromosomes 1, 4, 5, 7, 8, and 10. Chen et al. [38] found a significant resistance effect on chromosome 4. In the study by Li et al. [39], four QTLs related to resistance to fungi of the genus *Fusarium* were detected. They were located on chromosomes 3, 4, 5, and 6. The resistance allele in each of these four QTLs was transmitted by the resistant parent BT-1 and accounted for 2.5–10.2% of the phenotypic variation. The QTL with the largest effect detected on chromosome 4 can be treated as a *locus* of resistance to *Fusarium* in maize. Furthermore, as a result of GWAS, three ca ndidate genes were identified in these regions, which encode proteins belonging to the Glutaredoxin family, actin depolymerizing factors (ADFs), and AMP-binding proteins [40]. In 2016, Chen [39] and his colleagues presented 45 SNPs that were significantly associated with *Fusarium* resistance, each of which had a relatively small additive effect and explained 1–4% of the phenotypic variation [39].

Therefore, the primary objective of the research was to investigate the genetic mechanisms underlying maize resistance to fungi of the genus *Fusarium*. The authors, using NGS and association mapping, identified new silicoDArT and SNP markers linked to genes that confer resistance in maize plants to fungi of the genus *Fusarium*. Then, using physical mapping, the location of the markers and their linkage to nearby genes were determined. In the next step, primers were designed to identify the selected SilicoDArT and SNP markers linked to candidate genes that confer resistance to *Fusarium* in maize. The diagnostic procedures for identifying new molecular markers on reference materials susceptible and resistant to *Fusarium* were optimized. The expression level of selected candidate genes was also determined. Among those analyzed, potential genes that could carry maize resistance to fungi of the genus *Fusarium* were selected.

## 2. Results

### 2.1. Field Study

**Figure 1:**
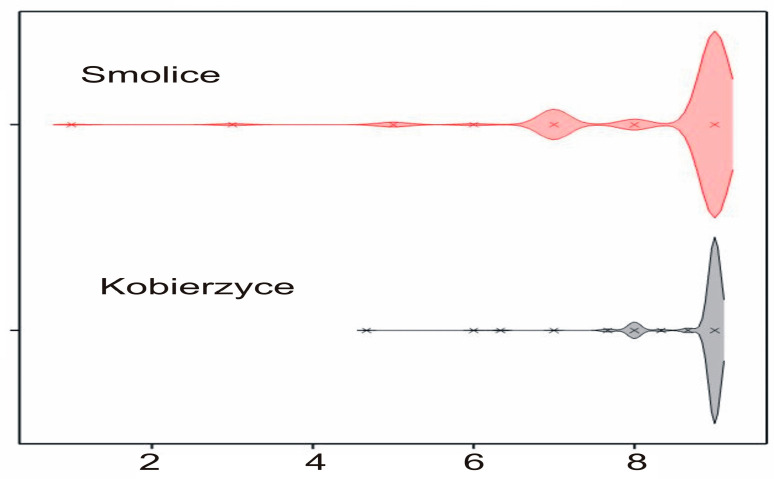
Density distribution of the degree of maize resistance to fungi of the genus *Fusarium*.

The field trial was established in two locations: Smolice (51° 42′ 58.904″ N 17° 13′ 29.13″ E) and Kobierzyce (50° 58′ 19.411″ N 16° 55′ 47.323″), with three replications, on 10 m^2^ plots, in the years 2021–2022. Throughout the entire growing season, the degree of maize plant infection by fungi of the genus *Fusarium* was assessed (Figure 1) by using the COBORU scale (Table 1). The density plot indicates that in Smolice, most genotypes had resistance levels of 7 and 9 (on a 9-point scale; 1—susceptible, 9—resistant), whereas in Kobierzyce, most genotypes were characterized by resistance levels of 8 and 9 (Figure 1).

### 2.2. Phenotyping

Supplementary Table S1 shows the mean values of maize infection by *Fusarium* fungi for individual hybrids at both locations simultaneously. The analysis of variance revealed significant differences in maize resistance to the pathogen, both among genotypes and between the locations where the field trial was conducted (Table 2). The observations were used for association mapping.

**Figure 2:**
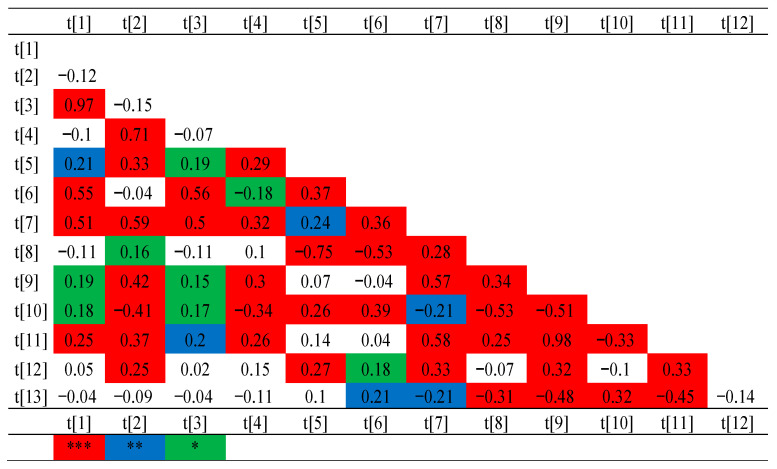
Correlations between the analyzed characteristics of the yield structure and the degree of infection by fungi of the genus *Fusarium* (in Smolice and Kobierzyce). 1-cob length, 2-cob diameter, 3-core length, 4-core diameter, 5-the number of rows of grain, 6-the number of grains in a row, 7-mass of grain from the cob, 8-1000-grain weight, 9-yield per plot, 10-dry matter content after harvest, 11-yield (t/ha), 12-resistance to fungi of the *Fusarium* of cob, 13-resistance to fungi of the *Fusarium* stem rot. *** *p* < 0.5, ** *p* < 0.01, * *p* < 0.001.

Additionally, correlations were analyzed between the degree of maize plant resistance to *Fusarium* fungi and the characteristics of yield and its structure at both locations (Smolice and Kobierzyce). The degree of resistance of maize plants by fungi of the genus *Fusarium* is significantly positively correlated with: the number of grain in a row (0.21), dry matter content after harvest (0.32) and significantly negatively correlated with: mass of grain from the cob (−0.21), 1000-grain weight (−0.31), yield per plot (−0.48), yield (−0.45) (Figure 2).

### 2.3. Assessment of Pathogenic Fungal Species

During the growing season, various maize kernel samples were collected and examined for colonization by *Fusarium*. The tested samples differed significantly in the degree of colonization by this pathogen. The most abundant fungi were *Fusarium poae* and *Fusarium culmorum.* Randomly selected cobs infected by fungal pathogens were stored to collect fungal spores, from which inoculum was prepared at a later stage of the experiment. These cobs were primarily infested with the following species: *F. graminearum*, *F. pseudograminerarum*, *F. boothii*, *F. subglutinans* (Table 3).

### 2.4. The Impact of Ostrinia nubilalis Hbn. on Maize Infection by Fungi of the Genus Fusarium

In 2021, on the maize plantation in Smolice, an average of 3.1% of maize plants were damaged by *Ostrinia nubilalis*, while in Kobierzyce, this pest damaged 3.8% of plants. A year later (2022), due to the feeding of *Ostrinia nubilalis*, 3.2% of maize plants were damaged in Smolice, while 2.4% were damaged in Kobierzyce. According to Baran et al. (2022), the areas of western and central Poland (where Smolice and Kobierzyce are located) are regions where the damage caused by this pest is relatively small compared to the southern regions, where in previous years the damage was recorded at up to 30% [41].

### 2.5. Genotyping and Association Mapping

**Figure 3:**
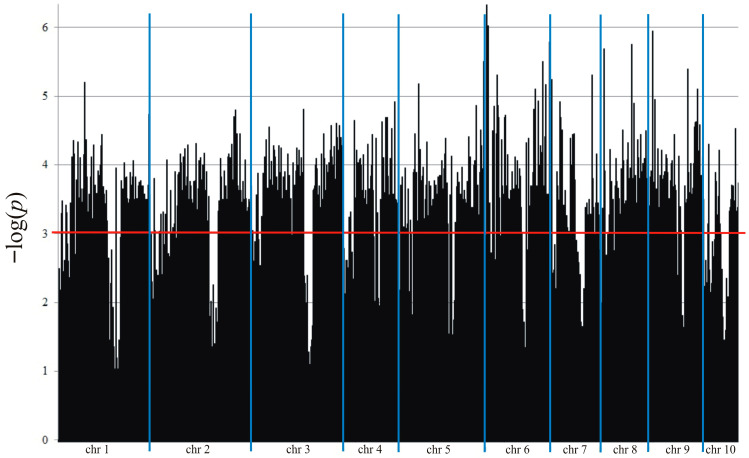
Manhattan plot for *Fusarium* per cobs.

As a result of NGS (next-generation sequencing), a total of 5714 significant SilicoDArT and SNP markers associated with maize resistance to *Fusarium* fungi were obtained (Figure 3). To determine the utility of the identified markers, MAF > 0.25 and a number of missing observations < 10% were applied (Table 4). The raw results from NGS are deposited on the Diversity Arrays Technology website at https://www.diversityarrays.com/, accessed on 1 May 2024 under the user account (Agnieszka Tomkowiak).

**Figure 4:**
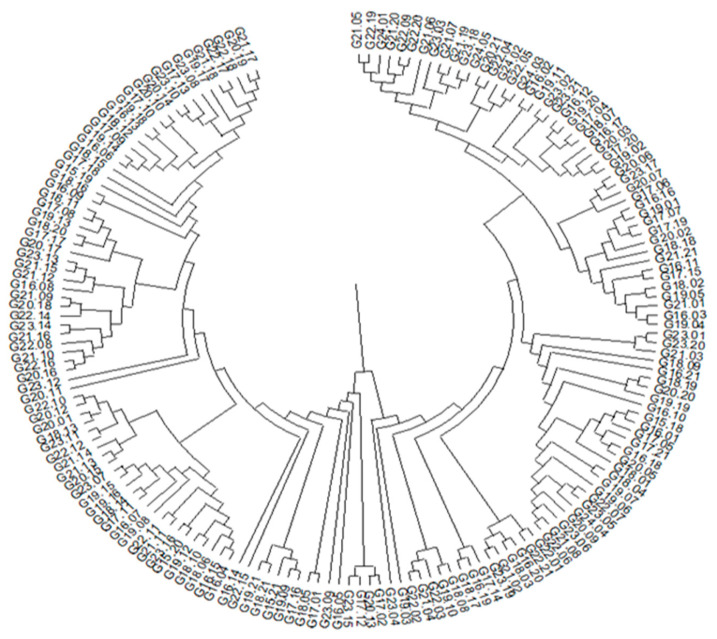
Dendrogram showing the genetic similarity between the analyzed genotypes.

Based on the identified SNP and SilicoDArT molecular markers, a dendrogram of genetic similarity was created for the 186 analyzed hybrids (Figure 2). The dendrogram reveals seven distinct similarity groups. The first group comprises 23 genotypes, numbered from G21.17 to G16.09, and the second group contains 21 genotypes, from G18.11 to G20.11. The third group consists of 27 genotypes ranging from G22.10 to G22.15, and the fourth group includes 29 genotypes, from G19.21 to G22.01. The fifth group comprises 26 genotypes, number ed from G22.06 to G23.01, the sixth group consists of 18 genotypes from G19.04 to G20.06. The seventh and last group includes 29 genotypes, from G19.02 to G21.05. These results can be used for selecting parental components for crosses, as it is most effective to choose genotypes from distinct genetic similarity groups to maximize genetic diversity (Figure 4).

### 2.6. Physical Mapping, Functional Gene Analysis

Out of the 5714 SilicoDArT and SNP markers significantly associated with maize resistance to *Fusarium*, 10 (with the highest LOD values) were selected based on their consistent significance across both testing locations—Kobierzyce and Smolice. An effort was also made to determine the physical locations of these selected markers within the maize genome (Table 5). Subsequently, primers were designed (Table 6) and used to identify the presence of the 10 selected markers in reference genotypes classified as either resistant or susceptible to *Fusarium* infection.

### 2.7. Gene Expression Analysis

**Figure 5:**
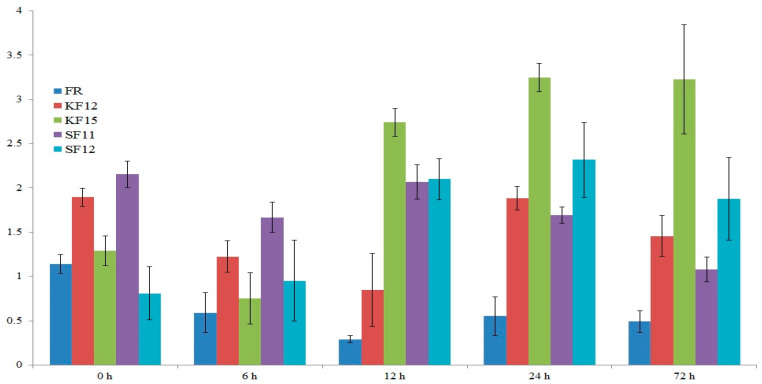
The expression level of the gene encoding for alpha-mannosidase I MNS5 before inoculation (0 h) and at 6, 12, 24, and 72 h after inoculation.

**Figure 6:**
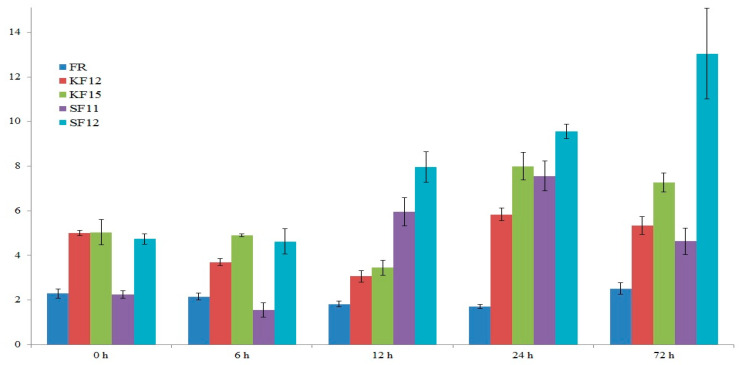
The expression level of the gene encoding for peroxisomal carrier protein before inoculation (0 h) and at 6, 12, 24, and 72 h after inoculation.

Gene expression was analyzed in leaves of five selected genotypes. Among them, four genotypes—KF12, KF15, SF11, and SF12—were highly resistant to *Fusarium* (rated 9 on a 9-point scale). As a control, a susceptible genotype to *Fusarium* (FR) was used (Figure 5 and Figure 6). The expression level was examined before inoculation (0 h) and at 6 h, 12 h, 24 h, as well as 72 h after inoculation (hai) Two genes whose function was preliminarily annotated (Table 6) were subjected to expression analysis. The first gene, which harbors the SNP 4583014 marker, is present on chromosome 5 and encodes alpha-mannosidase I MNS5 (Figure 5). The results of repeated-measures univariate analysis of variance indicate a statistically significant (*p* < 0.001) effect of line, term, and line term interaction on the expression of this gene. For the latter, at 6 hai, a decrease in expression level was observed in all varieties except SF12. At 12 hai, an increase in its expression level was observed in varieties KF15, SF11, and SF12. A further increase in the expression level of *alpha-mannosidase I MNS5* gene (24 hai) was observed for varieties KF15 and SF12. Finally, at 72 hai, the expression level of this gene increased in variety KF15, while in the other varieties it slightly decreased. Regarding the negative control (FR), a decrease in the expression level of the gene coding for alpha-mannosidase I MNS5 was noted at 6 hai. In subsequent hours, the expression level of this gene for the FR variety remained at a constant low level (Figure 5).

The second analyzed gene is located on chromosome 8, and encodes a peroxisomal carrier protein. This gene was selected due to the close proximity of the SilicoDArT 4579116 marker—just 240 base pairs away—making it a strong candidate for further investigation. The results of repeated-measures univariate analysis of variance indicate a statistically significant (*p* < 0.001) effect of line, term, and line term interaction on the expression of the *peroxisomal carrier* gene. The conducted qPCR analysis revealed a slight decrease in its expression level at 6 hai in all analyzed varieties. At 12 hai, an increase was seen in varieties SF11 and SF12. At 24 hai, the expression increased further in all varieties except the susceptible control (FR), which may indicate the activation of resistance processes. Finally, at 72 hai, an increase in the expression level of *peroxisomal carrier* gene was noted for the SF12 variety. The control (FR) did not show significant differences in the expression level of the analyzed gene at subsequent time intervals (Figure 6).

### 2.8. Transcriptomic Data Analysis

To further investigate the putative role of selected genes in maize response to fungal pathogens, an expanded analysis using available transcriptomic data was conducted. In the assessment, genes containing the analyzed markers, as well as those located within 500 base pairs of them, were considered (Table 5). Of the six analyzed genes, all exhibited more pronounced changes in expression levels in kernels 72 h after *Fusarium verticillioides* infection in the resistant genotype. In contrast, the changes in expression were less significant in the susceptible genotype (Table 7).

## 3. Discussion

In the context of the integrated plant protection paradigm, which has been in place for several years, and the ongoing reduction in the number of available pesticide active substances, cultivating disease-resistant varieties is becoming increasingly important. The European Green Deal policy, developed by the European Commission, aims to achieve climate neutrality, i.e., a net-zero reduction in greenhouse gas emissions, by 2050. It also aims to halve the use of chemical plant protection products by 2030. Combining classical breeding methods with molecular biology tools not only shortens the time required to breed new varieties but also contributes to reducing chemical protection through the cultivation of plants with increased, genetically determined disease resistance. One of the most serious maize diseases, causing significant yield losses, is Fusarium ear rot, caused by pathogenic fungi of the genus *Fusarium* spp.

The problem is not only the occurrence of the disease and the associated yield drop, but also the mycotoxins secreted by the fungus, which are harmful to humans and animals [43]. The climate warming in recent years (warmer growing seasons) causes increased contamination with mycotoxins, especially fumonisins and aflatoxins [44]. Therefore, the search for new maize protection strategies becomes crucial. The lack of effective fungicides means that breeding maize varieties resistant to *Fusarium* fungi is becoming increasingly important [45].

Many scientists emphasize the importance of understanding the genetic basis of the plant’s defense mechanism. Maize resistance to *Fusarium* fungi is a polygenic trait with complex inheritance [8,46]. Understanding the genetic mechanisms that control resistance to fungal diseases facilitates breeding. As early as 1994, Hoenisch and Davis [47] demonstrated that resistance to Fusarium ear rot is influenced by multiple genes, including those that affect the thickness of the pericarp and aleurone layers. Sampietro et al. [48] suggested that genes responsible for the production of wax and phenolic compounds are also involved in resistance processes. Netshifhefhe et al. [49] believe that plant resistance to *Fusarium* is the result of various interactions between genes. In our own research, it was shown that the degree of infestation of maize plants by *Fusarium* fungi was correlated with grain mass per ear, yield per plot, yield per ha, the number of rows, and ear diameter.

To specify the results of our analyses, the *Fusarium* ssp. fungi that occurred on infested maize kernels were also identified. The tested kernel samples of different maize genotypes differed significantly in terms of colonization by *Fusarium* fungi. The tested samples differed significantly in terms of colonization by this pathogen. The most common were *Fusarium poae* and *Fusarium culmorum* fungi. Single samples were very weakly colonized by *Fusarium* or not at all.

To understand the genetic architecture of polygenic traits, sequencing and analysis of entire plant genomes are becoming increasingly important. Next-generation sequencing (NGS) technology, thanks to its high efficiency and increasingly lower costs, has revolutionized modern biology [50]. This technology, combined with phenotyping and association mapping, enables the identification of various types of molecular markers and the genes linked to them, which condition important useful traits, including resistance to fungal diseases. Genome-wide association studies (GWAS) are a valuable tool for identifying candidate genes associated with quantitative traits. Zila et al. [35,36] used GWAS to identify markers related to maize resistance to *Fusarium*. The authors identified 10 SNP markers significantly associated with resistance to this pathogen. Zhou et al. [40], based on a genetic linkage map constructed using 1868 markers, identified 11 QTLs, including five stable QTLs related to resistance to *Fusarium* fungi. Transcriptome analysis allowed Zhang et al. [34] to identify 153 genes related to Fusarium ear rot resistance. Mitogen-activated protein kinase signaling pathways regulated the main resistance mechanisms of maize inbred lines to *F. graminearum* infection. Chen et al. [38] found that genes on chromosome 4 are responsible for *Fusarium* resistance. In the research by Li et al. [39], four QTLs were detected on chromosomes 3, 4, 5, and 6; according to the authors, the QTL detected on chromosome 4 can be treated as a locus for resistance to maize ear rot.

Through our own research, utilizing next-generation sequencing and association mapping, we identified a total of 5714 molecular markers related to maize plant resistance to *Fusarium* fungi. Of these, 10 markers showed significant association (with the highest LOD values), including both SNP and silicoDArT types, located on chromosomes 5, 6, 7, 8, and 9. Two markers, SNP 4583014 and SilicoDArT 4579116, were particularly noteworthy, since they are located within or in close proximity to pre-characterized genes, respectively. The SNP marker is located on chromosome 5, within exon 8 of the gene encoding *alpha-mannosidase I* MNS5. Recent studies indicate that biotic stresses, such as plant infestation by *Fusarium* fungi, can disrupt protein folding in the endoplasmic reticulum (ER). This process is vital for the cell, as newly formed polypeptide chains must fold into specific three-dimensional structures to function correctly. Misfolded proteins lose their functionality and will be degraded by regulatory mechanisms, such as chaperone proteins, sugar-binding lectins, and folding enzymes [51,52,53]. Strasser et al. [54] discovered the existence of the ERAD (ER-associated degradation) system in plants. Plants with defects in ERAD components show increased sensitivity to stress. According to Sun et al. [55], MNS4 and MNS5 are likely to be functionally distinct and play a crucial role in regulating ERAD and the endoplasmic reticulum stress response.

The second marker, SilicoDArT 4579116, is located 240 bp away from the gene on chromosome 8 encoding the peroxisomal carrier protein. Peroxisomes are eukaryotic organelles known for their dynamic morphology and metabolism. In plants, peroxisomes play crucial roles in numerous processes, including primary and secondary metabolism, development, and responses to abiotic and biotic stresses [56]. Major peroxisomal-dependent genes are associated with protein and endoplasmic reticulum (ER) protection at later stages of stress, while, at earlier stages, these genes are related to hormone biosynthesis and signaling regulation. Peroxisomal footprints provide a valuable resource for assessing and supporting key peroxisomal functions in cellular metabolism under both control and stress conditions across various species, including plants [57]. The clustered late peroxisome-dependent gene groups with regard to heat shock factors and proteins, as well as responses to ER stress and are mainly involved in protein protection and detoxification. Different transcription factors, in addition to hormone-dependent biosynthesis and signaling, mainly with respect to jasmonic acid, are present in early peroxisome-dependent genes; this suggests that initial peroxisomal stress may regulate different signaling pathways involved in plant responses to stress [58].

In our own research, the expression of *alpha-mannosidase I MNS5* and the *peroxisomal carrier* genes was analyzed in maize varieties following inoculation with *Fusarium*. The progression of *Fusarium* infection can be divided into two temporally distinct stages: an early stage, occurring between 12 and 48 h after-inoculation (hai), and a late stage, which begins at 72 hai. Therefore, expression analysis was conducted at different time points, reflecting the timeframe of the specified infection stages. In the qPCR assay, four genotypes, from two locations (Kobierzyce-K and Smolice-S), that are highly resistant (rated 9 on a 9-point scale) to *Fusarium*, were used and compared to a susceptible control genotype (FR). The obtained results showed statistically significant variation in expression depending on genotype, time after inoculation, and their interaction. Interestingly, at 6 h after-inoculation (hai), both genes showed a slight decrease in expression across nearly all tested genotypes, regardless of their resistance level. This phenomenon could be potentially associated with the early suppression of host defenses by *Fusarium* immediately upon infection. It has been shown that the plant pathogens, including *Fusarium*, produce effector proteins that interfere with host immune mechanisms, thereby facilitating infection and enhancing their virulence [59]. However, further investigation is needed to identify and characterize *Fusarium* effectors and to determine whether resistant maize genotypes counteract effector activity more effectively than susceptible ones.

From 12 hai onward, differences in gene expression began to emerge. The expression of *alpha-mannosidase I MNS5* increased in the resistant genotypes KF15 and SF12, while the *peroxisomal carrier* gene was upregulated in SF11 and SF12. At 24 hai, the expression of both genes continued to rise in the resistant lines, with KF15 and SF12 consistently showing elevated expression, which persisted through 72 hai. In contrast, the susceptible genotype FR maintained low expression levels of the two analyzed genes throughout the time course (Figure 5 and Figure 6).

These results highlight clear differences in gene expression dynamics between resistant and susceptible genotypes. The early (12 hai) and sustained upregulation of *alpha-mannosidase I MNS5* and *peroxisomal carrier* genes in the leaves of resistant lines suggests their involvement in the activation and maintenance of defense responses against *Fusarium* infection. This is further reinforced by an analysis of available transcriptomic data (Table 7), which shows more pronounced expression changes in six analyzed genes, including *alpha-mannosidase I MNS5* and the *peroxisomal carrier* gene, in the resistant genotype than in the susceptible one after *Fusarium verticillioides* infection. Although MNS5 and the peroxisomal carrier protein are potentially linked to stress-response pathways such as ERAD and JA/ROS signaling, there is currently no direct evidence in the literature connecting these genes to plant responses against *Fusarium* spp. This gap highlights the exploratory nature of our findings. By identifying markers within or near these genes and evaluating their expression patterns during infection, these results provide a valuable foundation for future functional studies aimed at confirming the roles of ERAD and peroxisome-mediated signaling in host–pathogen interactions.

Since marker-assisted selection (MAS) allows for reduced financial outlays and increased productivity, conditions for polymerase chain reaction (PCR) were developed to identify 10 significant molecular markers. As a result of the analyses, only one marker on agarose gels differentiated genotypes resistant and susceptible to *Fusarium* fungi. This marker can be used in practical breeding to select resistant genotypes. However, additional research is necessary to develop more accurate methods for distinguishing resistant and susceptible genotypes using the remaining markers. Conventional PCR combined with agarose gel electrophoresis may lack the sensitivity needed to detect subtle genetic differences such as single-nucleotide polymorphisms. Therefore, future studies will aim to improve detection techniques, including high-resolution melting (HRM) PCR, which offers better sensitivity and has already proven effective in genotype differentiation, as shown, for instance, in soybean rhizobia [60].

The molecular analyses carried out on maize genotypes focused not only on identifying new markers and QTL regions related to resistance to Fusarium fungi, but also on searching for methods that enable the selection of parental components for crosses to increase the yield of resistant varieties. This aspect, although discussed briefly in the publication, is also very important from a breeding perspective [61]. Therefore, an attempt was made to select parental components for crosses based on the genetic similarity between them, determined using the identified SilicoDArT and SNP molecular markers. According to literature reports, it is recommended to select resistant genotypes for crosses that have a large genetic distance between them.

## 4. Materials and Methods

### 4.1. The Plant Material

The plant material consisted of 186 F_1_ hybrids, which were submitted for next-generation sequencing. The F_1_ hybrids were obtained by crossing inbred lines belonging to different origin groups, characterized by varying resistance to *Fusarium* fungi (from 1 to 9 on the COBORU scale). Some of the parent lines were flint grain lines of three different origins: F_2_ (a group related to the F_2_ line, bred at INRA in France from the Lacaune population), EP_1_ (a group related to the EP_1_ line, bred in Spain from the population derived from the Pyrenees), and German Flint. The other part of the parent lines were dent-type kernels derived from various origin groups in the United States: Iowa Stiff Stalk Synthetic (BSSS), Iowa Dent (ID), and Lancaster. Five reference lines were used for gene expression analyses [four resistant to *Fusarium* (resistance level 9 on the COBORU scale) and one susceptible to *Fusarium* (resistance level 1 on the COBORU scale) as a negative control]. Field observations of these 5 lines were conducted for three years (2017–2020). A total of 191 genotypes were used in the experiment. Plant material came from two Polish breeding companies: Smolice Hodowla Roślin Sp. z o.o., Smolice, Poland and Małopolska Hodowla Roślin Sp. z o.o. from the IHAR Group Kobierzyce, Poland. Information on the origin of the parental forms of the F_1_ hybrids was also obtained from the breeding companies.

### 4.2. Phenotyping

#### 4.2.1. Field and Phytotron Experiments

This methodology was adopted from Sobiech et al. [62]. An experiment with 186 hybrids and 20 reference genotypes (susceptible and resistant to fungi of the genus *Fusarium*) was established on 10 m^2^ plots, in a randomized complete block design, with three replications, in two locations: Smolice (51° 42′ 58.904″ N 17° 13′ 29.13″ E) and Kobierzyce (50° 58′ 19.411″ N 16° 55′ 47.323″). Observations on the degree of infection of maize plants by fungi of the *Fusarium* genus were carried out at eight time points: time point 1—development of first kernels of watery consistency, containing approximately 16% dry matter (BBCH 71), time point 2—start of milky ripe stage of kernels (BBCH 73), time point 3—full milky ripe stage of kernels, containing approximately 40% dry matter (BBCH 75), time point 4—kernels reach typical size (BBCH 79), time point 5—start of dough stage of kernels, soft kernels containing approximately 45% dry matter (BBCH 83), time point 6—full dough stage of kernels, kernels with typical coloration containing approximately 55% dry matter (BBCH 85), time point 7—physiological maturity, visible black points at the base of the kernel containing approximately 60% dry matter (BBCH 87), time point 8—full maturity, hard and shiny kernels containing approximately 65% dry matter (BBCH 89). Observations were carried out on 20 randomly selected plants.

The COBORU scale, popular in Poland, was used to assess the degree of maize disease infection by *Fusarium* fungi. The Central Research Center for Cultivated Plant Varieties (COBORU) assesses the resistance of maize varieties to diseases and environmental factors using a nine-point scale. This scale is commonly used in post-registration testing (PDO) and has the following interpretation: a value of 9 indicates the variety has the highest resistance; a value of 1 indicates the variety has the lowest resistance or the highest susceptibility.

An experiment with five reference genotypes was conducted in a growth chamber under controlled conditions: the temperature was set at 22 °C during the day and 18 °C at night, with a 16 h photoperiod, and the relative humidity was maintained at 60 to 70%. Furthermore, the light source’s emission spectrum was set with a photon flux of 572 μE. Four maize kernels were sown in each 16 cm diameter pot with soil from a cultivated field, in four replications. The soil was maintained at approximately 70% of field capacity throughout the growth period. From the field where the maize experiment was conducted, cobs infected with Fusarium fungi were collected. Fungus samples were collected from the infected cobs to identify the specific species and prepare inoculum. The final species identified were *Fusarium graminearum* (isolate Fg/D), *Fusarium boothii* (isolates F0410/7, 20K), *Fusarium pseudograminearum* (isolates: F2811, 1428/12b), and *Fusarium subglutinans* (isolate ZK4). As it results from many years of observations conducted in Polish breeding companies, all *Fusarium* species that were used to prepare the inoculate are characterized by a similar degree of pathogenicity. Tetrazolium staining was used to assess spore viability. Spores were incubated with 0.1–0.5% TTC (or MTT) for several hours. Viable cells reduced tetrazolium to red or purple formazan. The entire assay was visually assessed. Next, a suspension containing all the fungal species presented above was prepared and suspended in water with 1% *v*/*v* Tween 20. The suspension contained approximately 5 × 10^5^ spores/mL and was prepared immediately before inoculation. Artificial infection was then performed by spraying plants at the 4–5 leaf stage. Each pot was sprayed with approximately 4 mL of inoculum. Leaf tissue fragments were collected at five time points: 0 h (before inoculation) and 6, 12, 24 and 72 h after inoculation in three biological replicates.

#### 4.2.2. Meteorological Conditions During the 2021 and 2022 Growing Seasons

Meteorological conditions during the 2021 growing season were favorable for maize growth and development, despite frost in April delaying sowing. The month of May, which is crucial for maize growth and development, was cool (12 °C) and wet, with a rainfall amount of 76 mm. In contrast to May, June and July 2021 turned out to be dry (June: 52.7 mm; July: 65 mm) and warm (June: 19.3 °C; July: 20.9 °C). The dry and warm weather did not favor the spread of fungal diseases during this period. In August, an increased infection of maize by *Fusarium* was observed, which was caused by a very high amount of rainfall (140.1 mm) and a fairly high temperature (17 °C). The very dry months of September (42.3 mm) and October (19.2 mm) inhibited the development of fungal diseases, including ear fusariosis. Given the above, all analyzed genotypes exhibited high resistance.

**Figure 7:**
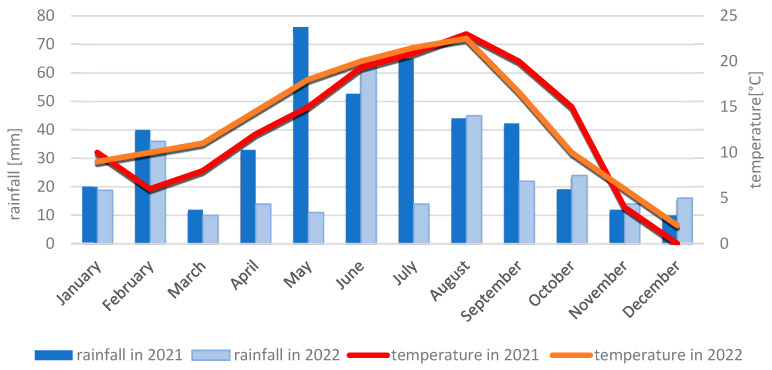
Average monthly temperature and average monthly precipitation in Smolice in 2021 and 2022. **(Sobiech)**.

**Figure 8:**
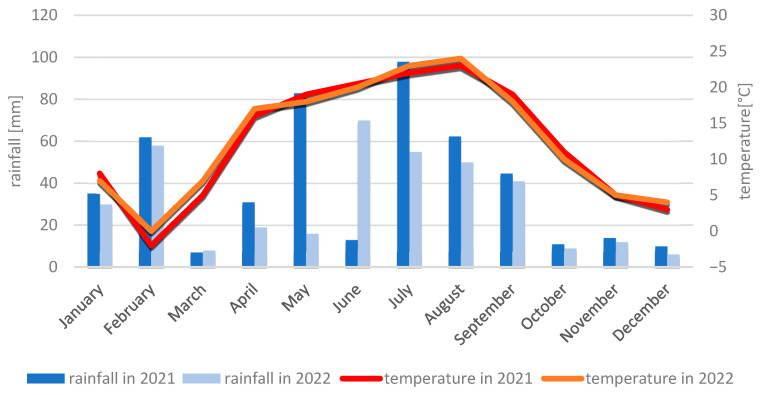
Average monthly temperature and average monthly precipitation in Kobierzyce in 2021 and 2022. **(Sobiech)**.

The 2022 growing season also favored the growth and development of maize in terms of meteorological conditions. May was a very warm and dry month, with an average monthly temperature of 15.2 °C and a monthly rainfall sum of only 10.8 mm. Low air humidity and a lack of major rainfall contributed to a deepening drought. The months of June, July, and August were characterized by an average temperature above the norm, 18.3 °C, 21.7 °C, and 22.1 °C, respectively. In contrast to May, June was the month with the highest rainfall sum in this period (63.4 mm), while July (13.3 mm) and August (44.8 mm) were very dry months. September was a warm month (16.7 °C) with little rainfall (22.5 mm), similar to October (10.2 °C, 24.6 mm). The dry and warm weather did not favor the development of fungal diseases throughout the maize growing season. In 2022, all analyzed genotypes were characterized by a resistance level of 6° to 9° on the COBORU scale. The meteorological conditions in Kobierzyce and Smolice in the years 2021–2022 are presented in Figure 7 and Figure 8.

#### 4.2.3. Influence of Ostrinia nubilalis Hbn. on Maize Infection by Fungi of the Genus Fusarium

At the end of August and the beginning of September 2021 and 2022, when the plants were in the full dough stage of kernels (BBCH 85) and damages caused by the pest were most visible, damages to maize by *Ostrinia nubilalis* Hbn. were analyzed. Three random points were chosen on each plot, and 10 consecutive plants were observed to look for signs of caterpillar feeding. The results were presented as the percentage of maize damage.

### 4.3. Genotyping

#### 4.3.1. DNA Isolation

DNA isolation from fragments of young leaf tissue was performed using the Maxwell^®^ RSC PureFood GMO reagent and Authentication Kit (Promega, Madison, WI, USA) according to the attached procedure. The concentration and purity of the isolated DNA samples were checked using a DeNovix DS-11 (Wilmington, NC, USA) spectrophotometer. Individual isolation efficiency was high, ranging from 120 ng/µL to 860 ng/µL depending on the genotype. The purity of individual samples was also very good, ranging from 1.7 to 2.0 at an absorbance of 260/280 and from 2.0 to 2.2 at an absorbance of 260/230. By obtaining a relatively high DNA concentration, the samples were adjusted to the concentration of 100 ng/µL required by Diversity Arrays Technology.

#### 4.3.2. Next-Generation Sequencing

Isolated DNA from the tested maize plants, in the amount of 25 µL at a concentration of 100 ng per genotype, was loaded onto two 96-well Eppendorf plates for analysis aimed at identifying SilicoDArT and SNP polymorphisms. The analyses were performed at Diversity Arrays Technology, University of Canberra in Australia, according to the methodology described in detail on the company’s website: (https://www.diversityarrays.com/technology-and-resources/dartseq/) (accessed on 1 May 2024).

DArTseq technology consists of the following steps:

1. DNA digestion with restriction enzymes—DNA is digested with two restriction enzymes: PstI (a “frequent” cutter recognizing G/C-rich sequences) and MseI (a “rare” cutter recognizing A/T-rich sequences). Using both enzymes yields DNA fragments of different lengths.
2. DNA library preparation—after digestion, adaptors are ligated to the DNA fragments (the PstI adaptor contains motifs enabling amplification and identification of PstI restriction fragments, while the MseI adaptor contains motifs enabling amplification and identification of MseI restriction fragments). Adaptors carry unique sequences (so-called barcodes) that allow multiple samples to be analyzed simultaneously on a single sequencer.
3. Amplification of fragment libraries—PCR is performed using primers complementary to the adaptors to create the fragment library to be sequenced. At this stage, selective PCR can be used with additional primers that include short sequences of chosen motifs to reduce the number of amplified fragments and increase specificity.
4. Selection and purification of fragments—PCR products are cleaned of excess primers and enzymes. These fragments can undergo further size selection (e.g., agarose gel or bead-based size selection), typically 250–500 bp, to ensure reproducibility.
5. High-throughput sequencing—using NGS platforms (Illumina), the fragments are sequenced en masse. The sequencing preparation kit includes adaptors and barcodes, enabling simultaneous sequencing of thousands to millions of fragments from many samples.
6. Bioinformatic analysis—sequences are processed and quality-filtered. Segments are compared to reference sequences or to each other to identify polymorphisms: SNPs (single nucleotide polymorphisms) and indels (insertions/deletions). A database of polymorphisms is generated, which can be used for various analyses, including genetic mapping.

#### 4.3.3. Association Mapping Using GWAS Analysis

Association mapping for plant resistance to *Fusarium* fungi of 186 F1 maize hybrids was performed using GWAS analysis. This mapping was conducted based on the results obtained from genotyping and phenotyping. Genotypic data were obtained from the DArTseq analysis, while the phenotypic data consisted of field results regarding the degree of plant infection by fungi of the genus *Fusarium*. For the association analysis, only SilicoDArT and SNP sequences meeting the following criteria were selected: one SilicoDArT and/or SNP within a given sequence (69 nt), minor allele frequency (MAF) > 0.25, and the missing observation fractions < 10%. Association mapping, based on SilicoDArT and SNP data and average trait values was conducted using the method based on the mixed linear model with a population structure estimated by eigenanalysis and modeled by random effects. All analyses and visualizations of the results were performed using procedure QSASSOCIATION (*GenStat* for Windows (10th Edition). QSASSOCIATION performs a mixed model marker–trait association analysis (also known as linkage disequilibrium mapping) with data from a single-environment trial. To avoid false positives in association mapping studies, some form of control is necessary for the genetic relatedness. The model used was specified by the RELATIONSHIPMODEL = eigenanalysis option, which infers the underlying genetic substructure in the population by retaining the most significant principal components from the molecular marker matrix; the scores of the significant axes are used as covariables in the mixed model, which effectively is an approximation to the structuring of the genetic variance covariance matrix by a coefficient of coancestry matrix (kinship matrix). Significance of association between *Fusarium* of cobs and SilicoDArT and SNP markers was assessed using *p* values corrected for multiple testing using the Benjamini–Hochberg method.

#### 4.3.4. Physical Mapping

The SilicoDArT and SNP marker sequences selected based on the GWAS analysis were subjected to a BLAST (Basic Local Alignment Search Tool) (https://blast.ncbi.nlm.nih.gov/Blast.cgi, accessed on 29 April 2025) analysis, which involves searching databases to find sequences with high homology. These analyses were performed on the URGI (Unité de Recherche Génomique Info) website with the fully sequenced maize genome. The URGI program was used to indicate the chromosomal locations of the found sequences similar to the analyzed sequences and to determine their physical location. To indicate the most probable region containing the most similar sequences to the analyzed ones, the combined probability was calculated for each chromosome based on the expected value (e-value). The sequences of all genes located in the designated area on the chromosome were subjected to further analysis.

#### 4.3.5. Functional Analysis of Gene Sequences

Functional analysis was performed using the Blast2GO program. (https://www.biobam.com/blast2go-basic/, accessed on 12 January 2025). The sequences of all genes located in the chromosome region were determined based on the BLAST analysis performed on the URGI website. The aim was to obtain information about the biological function of these genes.

#### 4.3.6. Designing Primers for Identified SilicoDArT and SNP Polymorphisms Associated with Maize Plant Resistance to Fungi of the Genus Fusarium

For each marker, pairs of polymerase chain reaction (PCR) primers were designed, with one primer covering the identified marker sequence and the other being complementary to the sequence adjacent to the analyzed marker.

### 4.4. mRNA Isolation

Materials were obtained from Sobiech et al. [62] RNA isolation from 75 reference plants (five genotypes × three biological replications × five time points), which were subjected to gene expression analysis, was conducted using a reagent kit from Promega (Maxwell^®^ RSC Plant RNA Kit). For expression analysis, plants were sampled before inoculation and 6 h, 12 h, 24 h, and 72 h after inoculation. A total of 75 samples were analyzed.

### 4.5. cDNA Synthesis

cDNA synthesis was performed using the iScript cDNA Synthesis Kit (Bio-Rad, Hercules, CA, USA). The process was carried out for 75 previously isolated mRNA samples. The reaction mixture included mRNA, H_2_O, and RT Supermix. The samples were incubated following this protocol: preparation (5 min at 25 °C), reverse transcription (20 min at 46 °C), and inactivation (1 min at 95 °C).

### 4.6. Gene Expression Analysis Using RT-qPCR

The methodology was adapted from Sobiech et al. [62]. RT-qPCR analyses were performed using iTaq Universal SYBR Green Supermix (Bio-Rad, Hercules, CA, USA) and a CFX96 Touch Real-Time PCR Detection System (Bio-Rad, Hercules, CA, USA). In accordance with the MIQE guidelines for the proper execution of real-time PCR analyses, three biological replicates were used for each gene at each time point for every sample tested. Additionally, three technical replicates were prepared for each of these. The obtained results were averaged. Furthermore, for each gene tested, a negative control without a cDNA template—NTC (No Template Control)—was also prepared, with three technical replicates for each test, similar to the tested genes. The composition of the RT-qPCR reaction mixture was as follows: iTaq supermix—5 μL, forward and reverse primers (10 μM)—0.5 μL each, 3 μL of nuclease-free water, and cDNA template—1 μL. The following temperature profile was used in the RT-qPCR reactions: initial denaturation for 3 min at 95 °C; followed by 40 cycles: denaturation for 10 s at 95 °C, primer annealing for 30 s at 60 °C, melting step (melting curve): temperature range from 65 °C to 90 °C; the temperature was increased by 0.5 °C every 5 s.

### 4.7. Reference Gene Analysis

The reference genes were obtained from the publication by Sobiech et al. [62]. Two reference genes were selected: β-tubulin and cyclophilin, based on the highest reaction efficiency (%E) values and determination coefficient (R^2^ > 0.997 for β-tubulin and R^2^ > 0.996 for cyclophilin). The applied temperature gradient allowed establishing 60 °C as the optimal primer annealing temperature during RT-qPCR for all analyzed genes. The results of the reference gene analysis were compared with the expression of candidate genes using the Gene Study tool (CFX Maestro, Bio-Rad Laboratories, Inc., Hercules, CA, USA). Primer sequences for the selected reference genes are shown below:

β-tubulin

5′CTACCTCACGGCATCTGCTATGT3′
3′AACACGAATCAAGCAGAG5′

Cyclophilin

5′CTGAGTGGTGGTCTTAGT3′
3′GTCACACACACTCGACTTCACG5′

### 4.8. Transcriptomic Analysis

Accession numbers used in transcriptomic data analysis were obtained from the National Center for Biotechnology Information (NCBI) website (www.ncbi.nlm.nih.gov, accessed on 15 September 2025). The transcriptomic data were from Lanubile et al. [42]. The changes in the expression level of the analyzed genes following *F. verticillioides* infection in the susceptible and resistant genotypes were calculated as a percentage of the expression value in the control. The expression level was evaluated in kernels 72 h after inoculation [42].

### 4.9. Evaluation of Pathogenic Fungal Species

During the growing season, fragments of maize plants showing symptoms of fusariosis were collected to isolate the pathogens causing the disease. The plant fragments were placed in 2% sodium hypochlorite for 1 min to disinfect the tissue surface. Subsequently, the plant fragments were placed on PDA medium (potato dextrose agar). After approximately 7 days, the grown cultures were transferred to a new medium. After preliminary selection of the obtained cultures for belonging to the genus *Fusarium*, cultivation on SNA medium was carried out, followed by microscopic evaluation of species affiliation. To confirm or verify the species affiliation of the obtained *Fusarium* isolates, the PCR technique was used. The ITS regions were amplified. The analysis concerned rDNA fragments bounded by DNA fragments complementary to the ITS1 (5′ TCCGTAGGTGAACCTGCGG 3′) and ITS4 (5′TCCTCCGCTTATTGATATGC3′) primers, as well as fragments of the *tef1* gene (encoding the transcription elongation factor) and *β-tub* (encoding β-tubulin). The obtained DNA fragments were then sequenced. The BLASTn program (http://blast.ncbi.nlm.nih.gov/, accessed on 2 July 2025.) and FUSARIUM-ID v. 1.0, a publicly available database of partial translation elongation factor 1-alpha (TEF) DNA, were used for sequence identification.

### 4.10. Statistical Analyses

The conformity of the empirical distributions of observed trait with the normal distribution was assessed using the Shapiro–Wilk *W*-test [63]. Homogeneity of variances was evaluated using Bartlett’s test. Two-way analysis of variance (ANOVA) was conducted to assess the effect of genotypes, location, and genotype × location interaction on the value of the observed trait. The relationships between observed traits were assessed using Pearson’s linear correlation coefficient calculated from genotype mean values. Genetic similarity between analyzed genotypes was calculated based on Nei and Li. The obtained genetic similarity coefficients were used for the hierarchical clustering of genotypes using the unweighted pair group method with the arithmetic mean (UPGMA). Association mapping was conducted based on species mean trait values and the generated marker data, using a mixed linear model (MLM) approach. This model incorporated population structure inferred via eigen analysis and modeled as random effect [64]. All statistical analyses and result visualizations were carried out using Genstat 23.1 software [65] specifically employing the QSASSOCIATION procedure [66,67]. For comparisons of time measurements of alpha-mannosidase I MNS5 and peroxisomal carrier protein genes, a repeated measures ANOVA was performed.

## 5. Conclusions

Current achievements in plant biotechnology surpass previous expectations, and the prospects for their application are even more promising. Furthermore, a deeper understanding of plant biology, facilitated by the use of “omics” technologies, molecular biology resources, and advanced data analysis platforms, has been translated into agricultural practice and enabled the improvement of many crop species. In light of the above, the intensification of agricultural development requires efficient technologies for producing economically viable and competitive plant products resistant to biotic stresses. In the study, a total of 5714 molecular markers related to the resistance of maize plants to fungi of the genus *Fusarium* were identified using next-generation sequencing and association mapping. Of these markers, 10 were selected that were significantly associated with the disease. These markers were identified on chromosomes 5, 6, 7, 8, and 9. The authors were particularly interested in two markers: SNP 4583014 and SilicoDArT 4579116. The SNP marker is located on chromosome 5, in exon 8 of the gene encoding alpha-mannosidase I MNS5. The SilicoDArT 4579116 marker is located 240 bp from the peroxisomal carrier protein gene on chromosome 8. Our own research and the presented literature review indicate that both these genes may be involved in biochemical reactions triggered by stress caused by the infection of plants with spores of *Fusarium* fungi. Analysis of the expression of both genes confirmed their role in resistance processes, as resistant varieties responded with an increase in the expression level of these genes at various time points after being inoculated with spores of *Fusarium* fungi. In the case of the control, which was susceptible to *Fusarium*, no significant fluctuations in the expression level of either gene were observed. Analyses concerning the identification of *Fusarium* fungi showed that the most abundant fungi on the infected maize kernels were *Fusarium poae* and *Fusarium culmorum*. Single samples were very poorly colonized by *Fusarium* or not at all. By using various molecular technologies, we have identified genomic regions associated with maize resistance to fungi of the genus *Fusarium*, which is of fundamental importance for understanding these regions and potentially manipulating them.

## Figures and Tables

**Table 1:** Interpretation of the COBORU scale used to assess ear colonization by *Fusarium* fungi.

Infection Degree	Description
9	clean ears, without mycelium
7	isolated fungal colonies on the cobs
5	50% of the cobs colonized by mycelium
3	75% of the cobs colonized by mycelium
1	entire cobs colonized by mycelium

**Table 2:** Mean squares from the analysis of variance for the degree of maize infection by fungi of the genus *Fusarium*.

Source of Variability	Hybrid	Residual
Number of degrees of freedom	172	346
Cob fusariosis [1,2,3,4,5,6,7,8,9]	0.393 ***	0.2004

*** *p* < 0.001.

**Table 3:** Heatmap presenting the colonization of maize kernels by fungi, particularly those of the genus *Fusarium*.

	% Colonization of Kernels by Fungi							% Colonization of Kernels by Fungi					
Sample	*F. poae*	*F. culmorum*	*F. graminearum*	*F. pseudograminerarum*	*F. boothii*	*F. subglutinans*	Sample	*F. poae*	*F. culmorum*	*F. graminearum*	*F. pseudograminerarum*	*F. boothii*	*F. subglutinans*
G21.08	**45**	**0**	**0**	**0**	**0**	**8**	G23.03	**24**	**8**	**0**	**21**	**0**	**0**
G21.09	**4**	**11**	**0**	**7**	**0**	**0**	G23.04	**19**	**12**	**0**	**0**	**0**	**0**
G21.10	**64**	**9**	**5**	**0**	**0**	**0**	G23.05	**61**	**0**	**0**	**0**	**0**	**0**
G21.11	**23**	**21**	**0**	**8**	**0**	**1**	G23.06	**38**	**0**	**0**	**0**	**0**	**0**
G21.12	**16**	**6**	**0**	**0**	**12**	**6**	G23.07	**14**	**0**	**0**	**0**	**0**	**1**
G21.13	**7**	**8**	**0**	**0**	**0**	**0**	G23.08	**45**	**0**	**0**	**0**	**0**	**1**
G21.14	**27**	**14**	**0**	**0**	**0**	**0**	G23.09	**12**	**0**	**0**	**0**	**0**	**9**
G21.15	**78**	**9**	**0**	**5**	**0**	**0**	G23.10	**54**	**1**	**0**	**16**	**0**	**9**
G21.16	**41**	**15**	**0**	**0**	**0**	**0**	G23.11	**6**	**0**	**0**	**0**	**0**	**1**
G21.17	**9**	**10**	**0**	**0**	**0**	**12**	G23.12	**1**	**0**	**0**	**0**	**0**	**1**
G21.18	**12**	**6**	**0**	**0**	**0**	**11**	G23.13	**1**	**0**	**0**	**0**	**0**	**3**
G21.19	**15**	**8**	**0**	**4**	**25**	**7**	G23.14	**8**	**0**	**0**	**0**	**0**	**0**
G21.20	**32**	**2**	**0**	**0**	**0**	**9**	G23.15	**34**	**21**	**0**	**0**	**0**	**0**
G21.21	**12**	**1**	**0**	**0**	**0**	**12**	G15.21	**38**	**12**	**0**	**0**	**23**	**0**
G22.01	**6**	**6**	**0**	**0**	**28**	**3**	G16.01	**12**	**16**	**0**	**0**	**0**	**0**
G22.02	**1**	**9**	**0**	**0**	**0**	**0**	G16.02	**17**	**9**	**0**	**0**	**0**	**4**
G22.03	**37**	**22**	**0**	**12**	**0**	**0**	G16.03	**26**	**9**	**6**	**0**	**0**	**4**
G22.04	**29**	**27**	**0**	**0**	**0**	**0**	G16.04	**3**	**14**	**0**	**0**	**0**	**4**
G22.05	**8**	**19**	**0**	**0**	**0**	**0**	G16.05	**3**	**11**	**0**	**0**	**17**	**1**
G22.06	**7**	**7**	**0**	**0**	**0**	**0**	G16.06	**45**	**6**	**0**	**0**	**0**	**1**
G22.07	**12**	**21**	**0**	**1**	**8**	**2**	G16.07	**39**	**2**	**0**	**0**	**0**	**0**
G22.21	**38**	**9**	**0**	**0**	**0**	**1**	G16.08	**22**	**15**	**0**	**0**	**0**	**0**
G23.01	**45**	**14**	**0**	**0**	**0**	**11**	G16.09	**12**	**7**	**0**	**0**	**0**	**0**
G23.02	**29**	**8**	**0**	**0**	**0**	**0**	G16.10	**16**	**8**	**1**	**0**	**0**	**0**

The lighter the red color, the lighter the value.

**Table 4:** Molecular markers SilicoDArT and SNP significantly associated with maize resistance to Fusarium fungi in the localities of Kobierzyce and Smolice (significant associations selected at *p* < 0.001 with Benjamini–Hochberg correction for multiple testing).

Trait	Number of SilicoDArT and SNP Markers	Effect (Min.)	Effect (Max.)	Effect (Average)	Percentage of Explained Variance (Min.)	Percentage of Explained Variance (Max.)	Percentage of Explained Variance (Average)	LOD (Min.)	LOD (Max.)	LOD (Average)
Cob fusariosis [scale 1–9]	5714	−0.759	0.2999	−0.016	1.7	13.3	4.65	1.3	6.33	2.59

**Table 5:** Characteristics and location of markers significantly associated with maize resistance to *Fusarium* fungi.

Marker Number (Marker Type)	Marker Effect (Percentage Variance Accounted for)	Chromosomal Localization	Marker Sequence (5′-3′)	Neighboring Genes
14586781 (SilicoDArT)	0.2999 (13.3%)	Chr6, 145045072 bp	TGCAGAATAAAGGCCGTAGCTACTAGCATGAGATCGGAAGAGCGGTTCAGCAGGAATGCCGAGACCGAT	312 bp downstream uncharacterized LOC100277697 29 kbp downstream LOC103631461 lysine-specific demethylase JMJ18 59 kbp downstream LOC109940629 ABC transporter G family member 45-like 89 kbp upstream uncharacterized LOC100276712
7049252 (SilicoDArT)	0.2871 (12.6%)	Chr6, 145473013 bp	TGCAGAGCAGAAGCCTTCCGCTGAAACGAGCCGGCCAGCCGGGTCAAAGCGGCGGGCGAATGCATGAGA	6.5 kbp upstream LOC100282707 Phosphoribosylaminoimidazole-succinocarboxamide synthase 52 kbp upstream uncharacterized LOC100281684 47 kbp downstream LOC100286030 ovate protein 527 kbp downstream LOC103630162 ATP-dependent DNA helicase DDX11
4778172 (SilicoDArT)	0.2744 (12.5%)	Chr9, 62605002 bp	TGCAGTCTCCAGCCGGCAGTGGCTGCGAACCAGTGACGAGATGAGCACGTCATCTGAAGGTCCCTCCTG	In intron of uncharacterized LOC100274139 54 kbp upstream LOC103638554 thioredoxin domain-containing protein PLP3A-like 173 kbp upstream LOC103638555 protein ALP1-like
2414058 (SNP)	0.2753 (12.1%)	Chr6, 151006006 bp	TGCAGCACACCTTCAAACCGTTTCCCCTCTAAACTGGCAAGATCATTGCATAGATCAGCAATACAAGAC	In exon 8 of uncharacterized LOC100280235 8 kbp upstream uncharacterized LOC100284086 13 kbp upstream uncharacterized LOC100284524 15 kbp downstream uncharacterized LOC109940566
4579116 (SilicoDArT)	−0.2759 (12.0%)	Chr8, 116991138 bp	TGCAGGCTGAAGCCGTTCCGGAAGGCATACCAAACTGATTCATACCAAACTTTGAGGCATGAGATCGGA	240 bp downstream LOC100282644 peroxisomal carrier protein 8.8 kbp upstream uncharacterized LOC100272776 49 kbp upstream LOC103637197 E3 ubiquitin-protein ligase AIRP2 54 kbp downstream uncharacterized LOC103635741
4587705 (SilicoDArT)	0.2660 (11.9%)	Chr8, 170156900 bp	TGCAGTAGCCTCGTCGTCACCGACATAACCTGAAAAAATCATTCAATTGACTCATGTAGTAGCGCCCCC	In exon 3 of uncharacterized LOC100272376 6.3 kbp downstream uncharacterized LOC100191367 18.8 kbp downstream LOC100383455 U-box domain-containing protein 7 27 kbp downstream LOC103636398 phospholipase A1 EG1, chloroplastic/mitochondrial
25947704 (SNP)	0.2586 (11.4%)	Chr6, 148365780 bp	TGCAGCAACGAGGCGGAGGAGGAGGCCGGGTTCAACCTCCTGGGGCTGCTGGTCGCCGCCATCATCGCG	In exon 1 of uncharacterized LOC118472251 2.6 kbp upstream LOC100273680 F-box family protein 5.5 kbp downstream uncharacterized LOC103630208 20 kbp upstream LOC103630207 GRAS family protein TF80
4583014 (SNP)	0.2586 (11.4%)	Chr5, 214247330 bp	TGCAGCTTCATATCTAGAATCACCAGTCAAACGTGACAACACACCCATTTCAAGTATAAGGGAACCTGT	In exon 8 of LOC103627708 alpha-mannosidase I MNS5 7 kbp downstream uncharacterized LOC100277687 7 kbp upstream LOC100284887 peroxidase 65 10.9 kbp upstream uncharacterized LOC100382440
4777510 (SNP)	0.2668 (11.2%)	Chr9, 117791088 bp	TGCAGATGAATAAATATTAGATATATTGACAACTTAAGTATCTGAGTGGCGCAAATTGAAGTTCTGATC	In intron 2 of uncharacterized LOC103652964 47 bp downstream uncharacterized LOC100191353 48 kbp downstream LOC103640211 uncharacterized sugar kinase slr0537-like 90 kbp upstream uncharacterized LOC103638900
4584918 (SNP)	0.2574 (11.0%)	Chr7, 144651551 bp	TGCAGTGCTCTAGGAACTTGGTTCTTCTCAGTTGCGGGTGCTCTTGTTGCTATTCCTGTGGGCATAAAG	In exon 2 of uncharacterized LOC103633003 5.3 kbp downstream uncharacterized LOC103633004 14.5 kbp downstream uncharacterized LOC100282132 37 kbp downstream LOC100283686 CHY1 60 kbp upstream LOC100281183 ATP-dependent RNA helicase DRS1

**Table 6:** Sequences of the designed primers used to identify newly selected markers significantly associated with maize resistance to *Fusarium* fungi.

Marker Number	Primer Sequences	PCR Product (bp)
**4586781**	**Forward:** ACAAAAGCTCTATAAATCTCTTAAA **Reverse:** CAAATATTCAGTAGTAAAGGATATC	218
**7049252**	**Forward:** GCGTCTCATGCATTCGCAC **Reverse:** CTACACTCAAGCAACTAAGGTCATC	235
**4778172**	**Forward:** GCGAACCAGTGACGAGATGAGCAAG **Reverse:** GGTCCTAGTCGGTCCCTGGTCG	258
**2414058**	**Forward:** ATCATTGCATAGATCAGCAATACCG **Reverse:** ATTCGTTGTATCAAGTGAAAACGCT	490
**4579116**	**Forward:** TTTGATATGGCTCCTGCAAG **Reverse:** GAATAAGGTGTGTATCTGGGG	489
**4587705**	**Forward:** CGTCACCGACATAACCTGAAAAAGT **Reverse:** TTTTCTTAAGGATTCTGCCACAATC	210
**25947704**	**Forward:** CACATGCTGAAGCTGATCCGAAACC **Reverse:** AGGAGGAGGCCGGGTTCAACCGT	241
**4583014**	**Forward:** CTATCAGCTAAAATGATAAGAATG **Reverse:** CCATTTCAAGTATAAGGGCG	321
**4777510**	**Forward:** GCAGCCAACAAATCCATC **Reverse:** AAGTTGTCAATATGTCTAATACG	309
**4584918**	**Forward:** GGCTCGGTGGAGTCAGCTTGTG **Reverse:** ATAGCAACAAGAGCACCCGCAACCG	200

**Table 7:** The heatmap illustrates the percentage changes in gene expression caused by *F. verticillioides* infection compared to the non-infected control. For any given gene, red signifies the genotype exhibiting a more pronounced change, while green indicates the genotype with a less pronounced change. Data from Lanubile et al. (2014) [42].

Marker	Gene Locus	Accession Number	*F. verticillioides* Infection	
			Susceptible Genotype	Resistant Genotype
4778172	LOC100274139	GRMZM2G093598	−6.4%	+7.5%
4579116	LOC100282644	GRMZM2G149211	+73.8%	+126.2%
4587705	LOC100272376	GRMZM5G805585	−11.4%	+59.4%
4583014	LOC103627708	GRMZM2G501450	+12.11%	−29.5%
4777510	LOC103652964	GRMZM2G162859	−2.4%	−14.4%
4584918	LOC103633003	GRMZM5G854301	+9.3%	+66.3%

## Data Availability

The original contributions presented in this study are included in the article/Supplementary Material. Further inquiries can be directed to the corresponding author(s).

## Supplementary Materials

The following supporting information can be downloaded at: https://www.mdpi.com/article/10.3390/ijms262110534/s1.

## References

[1] Erenstein O., Jaleta M., Sonder K., Mottaleb K., Prasanna B.M. (2022). Global maize production, consumption and trade: Trends and R&D implications. Food Secur..

[2] Zhang H., Lu Y., Ma Y., Fu J., Wang J. (2021). Genetic and molecular control of grain yield in maize. Mol. Breed..

[3] Zhu M., Tong L., Xu M., Zhong T. (2021). Genetic dissection of maize disease resistance and its applications in molecular breeding. Mol. Breed..

[4] Perry E.D., Moschini G. (2020). Neonicotinoids in US maize: Insecticide substitution effects and environmental risk. J. Environ. Econ. Manag..

[5] Ji D., Li L., Wang Y., Zhang J., Cheng M., Sun Y., Liu Z., Wang L., Tang G., Hu B. (2014). The heaviest particulate air-pollution episodes occurred in northern China in January, 2013: Insights gained from observation. Atmos. Environ..

[6] Deng B., Carter R.A., Cheng Y., Liu Y., Eddy L., Wyss K.M., Ucak-Astarlioglu M.G., Luong D.X., Gao X., JeBailey K. (2023). High-temperature electrothermal remediation of multi-pollutants in soil. Nat. Commun..

[7] Ranum P., Peña-Rosas J.P., Garcia-Casal M.N. (2014). Global maize production, utilization, and consumption. Ann. New York Acad. Sci..

[8] Butrón A., Reid L.M., Santiago R., Cao A., Malvar R.A. (2015). Inheritance of maize resistance to gibberella and fusarium ear rots and kernel contamination with deoxynivalenol and fumonisins. Plant Pathol..

[9] Scauflaire J., Mahieu O., Louvieaux J., Foucart G., Renard F., Munaut F. (2011). Biodiversity of *Fusarium* species in ears and stalks of maize plants in Belgium. Eur. J. Plant Pathol..

[10] Bode W.M., Calvin D.D. (1990). Yield-loss relationships and economic injury levels for European corn borer (*Lepidoptera*: *Pyralidae*) populations infesting Pennsylvania field corn. J. Econ. Entomol..

[11] Szőke C., Zsubori Z., Pók I., Rácz F., Illés O., Szegedi I. (2002). Significance of the European corn borer (*Ostrinia nubilalis* Hübn.) in maize production. Acta Agron. Hung..

[12] Blandino M., Reyneri A., Vanara F., Tamietti G., Pietri A. (2009). Influence of agricultural practices on *Fusarium* infection, fumonisin and deoxynivalenol contamination of maize kernels. World Mycotoxin J..

[13] Sobek E.A., Munkvold G.P. (1999). European corn borer (*Lepidoptera*: *Pyralidae*) larvae as vectors of *Fusarium moniliforme*, causing kernel rot and symptomless infection of maize kernels. J. Econ. Entomol..

[14] Garcia-Ceron D., Lowe R.G.T., McKenna J.A., Brain L.M., Dawson C.S., Clark B., Berkowitz O.P., Whelan J., Bleackley M.R., Anderson M.A. (2021). Extracellular Vesicles from *Fusarium graminearum* Contain Protein Effectors Expressed during Infection of Corn. J. Fungi.

[15] Ncube E., Truter M., Flett B.C., Van Den Berg J., Erasmus A., Viljoen A. (2020). Fungal mycoflora associated with *Busseola fusca* frass in maize plants. Afr. Entomol..

[16] Vigier B., Reid L.M., Dwyer L.M., Stewart D.W., Sinha R.C., Arnason J.T., Butler G. (2001). Maize resistance to Gibberella ear rot: Symptoms, deoxynivalenol, and yield. Can. J. Plant Pathol..

[17] Mesterházy Á., Lemmens M., Reid L.M. (2012). Breeding for resistance to ear rot caused by *Fusarium* spp. in maize—A review. Plant Breed..

[18] Uwe L., Miedaner T., Bürstmayr H., Vögele R.T. (2020). Breeding for Resistance to *Fusarium* Ear Diseases in Maize and Small-Grain Cereals Using Genomic Tools. Ph.D. Thesis.

[19] Sansaloni C., Petroli C., Jaccoud D., Carling J., Detering F., Grattapaglia D., Kilian A. (2011). Diversity Arrays Technology (DArT) and next-generation sequencing combined: Genome-wide, high throughput, highly informative genotyping for molecular breeding of *Eucalyptus*. BMC Proc..

[20] Elshire R.J., Glaubitz J.C., Sun Q., Poland J.A., Kawamoto K., Buckler E.S., Mitchell S.E. (2011). A robust, simple genotyping-by-sequencing (GBS) approach for high diversity species. PLoS ONE.

[21] Courtois B., Audebert A., Dardou A., Roques S., Ghneim-Herrera T., Droc G., Frouin J., Rouan L., Gozé E., Kilian A. (2013). Genome-Wide Association Mapping of Root Traits in a Japonica Rice Panel. PLoS ONE.

[22] Cruz V.M.V., Kilian A., Dierig D.A. (2013). Development of DArT Marker Platforms and Genetic Diversity Assessment of the U.S. Collection of the New Oilseed Crop *Lesquerella* and Related Species. PLoS ONE.

[23] Sakhare A.S., Kota S., Rathod S., Parmar B., Chinnusamy V. (2022). Genome-Wide Association Study. Genotyping by Sequencing for Crop Improvement.

[24] Abdurakhmonov I.Y., Abdukarimov A. (2008). Application of Association Mapping to Understanding the Genetic Diversity of Plant Germplasm Resources. Int. J. Plant Genom..

[25] Rafalski J.A. (2010). Association genetics in crop improvement. Curr. Opin. Plant Biol..

[26] Zhu C., Gore M., Buckler E.S., Yu J. (2008). Status and Prospects of Association Mapping in Plants. Plant Genome J..

[27] Rakoczy-Trojanowska M., Krajewski P., Bocianowski J., Schollenberger M., Wakuliński W., Milczarski P., Masojć P., Targońska-Karasek M., Banaszak Z., Banaszak K. (2017). Identification of Single Nucleotide Polymorphisms Associated with Brown Rust Resistance, α-Amylase Activity and Pre-harvest Sprouting in Rye (*Secale cereale* L.). Plant Mol. Biol. Rep..

[28] Edwards D., Batley J., Snowdon R.J. (2013). Accessing complex crop genomes with next-generation sequencing. Theor. Appl. Genet..

[29] Bar-Hen A., Charcosset A., Bourgoin M., Guiard J. (1995). Relationship between genetic markers and morphological traits in a maize inbred lines collection. Euphytica.

[30] Pritchard J.K. (2001). Deconstructing Maize Population Structure. Nat. Genet..

[31] Lanubile A., Pasini L., Lo Pinto M., Battilani P., Prandini A., Marocco A. (2011). Evaluation of broad spectrum, sources of resistance to *Fusarium verticillioides* and advanced maize breeding lines. World Mycotoxin J..

[32] Clements M.J., Kleinschmidt C.E., Maragos C.M., Pataky J.K., White D.G. (2003). Evaluation of inoculation techniques for *Fusarium* ear rot and fumonisin contamination of corn. Plant Dis..

[33] Zhang F., Zhang F., Wan X.Q., Pan G.T. (2007). Molecular mapping of QTL for resistance to maize ear rot caused by *Fusarium moniliforme*. Agric. Sci. China.

[34] Zhang X., Zheng S., Yu M., Xu C., Li Y., Sun L., Qiu X. (2024). Evaluation of Resistance Resources and Analysis of Resistance Mechanisms of Maize to Stalk Rot Caused by *Fusarium graminearum*. Plant Dis..

[35] Zila C.T., Ogut F., Romay M.C., Gardner C.A., Buckler E.S. (2014). Genome wide association study of *Fusarium* ear rot disease in the U.S.A. maize inbred line collection. BMC Plant Biol..

[36] Zila C.T., Samayoa L.F., Santiago R., Butrón A., Holland J.B. (2013). A genome-wide association study reveals genes associated with fusarium ear rot resistance in a maize core diversity panel. G3 Genes Genomes Genet..

[37] De Jong G., Pamplona A.K.A., Von Pinho R.G., Balestre M. (2018). Genome-wide association analysis of ear rot resistance caused by *Fusarium verticillioides* in maize. Genomics.

[38] Chen J., Ding J., Li H., Li Z., Sun X., Li J., Dai X., Dong H., Song W., Chen W. (2012). Detection and verification of quantitative trait loci for resistance to *Fusarium* ear rot in maize. Mol. Breed..

[39] Li Z.M., Ding J.Q., Wang R.X., Chen J., Sun X., Chen W., Song W., Dong H., Dai X., Xia Z. (2011). A new QTL for resistance to *Fusarium* ear rot in maize. J. Appl. Genet..

[40] Wu Y., Zhou Z., Dong C., Chen J., Ding J., Zhang X., Mu C., Chen Y., Li X., Li H. (2020). Linkage mapping and genome-wide association study reveals conservative QTL and candidate genes for *Fusarium* rot resistance in maize. BMC Genom..

[41] Baran M., Roik K., Bocianowski J. (2022). Analysis of monitoring of maize damage caused by the European corn borer (*Ostrinia nubilalis* Hbn.) in Poland in 2006–2016. Prog. Plant Prot..

[42] Lanubile A., Ferrarini A., Maschietto V., Delledonne M., Marocco A., Bellin D. (2014). Functional genomic analysis of constitutive and inducible defense responses to *Fusarium verticillioides* infection in maize genotypes with contrasting ear rot resistance. BMC Genom..

[43] Escrivá L., Font G., Manyes L. (2015). In vivo toxicity studies of fusarium mycotoxins in the last decade: A review. Food Chem. Toxicol..

[44] Yuan G., Shi J., Zeng C., Shi H., Yang Y., Zhang C., Shen Y. (2024). Integrated analysis of transcriptomics and defense-related phytohormones to discover hub genes conferring maize Gibberella ear rot caused by *Fusarium Graminearum*. BMC Genom..

[45] Mesterhazy A. (2024). Food Safety Aspects of Breeding Maize to Multi-Resistance against the Major (*Fusarium graminearum*, *F. verticillioides*, *Aspergillus flavus*) and Minor Toxigenic Fungi (*Fusarium* spp.) as Well as to Toxin Accumulation, Trends, and Solutions—A Review. J. Fungi.

[46] Martin M., Dhillon B.S., Miedaner T., Melchinger A.E. (2012). Inheritance of resistance to gibberella ear rot and deoxynivalenol contamination in five flint maize crosses. Plant Breed..

[47] Hoenisch R.W., Davis R.M. (1994). Relationship between kernel pericarp thickness and susceptibility to *Fusarium* ear rot in field corn. Plant Dis..

[48] Sampietro D.A., Vattuone M.A., Presello D.A., Fauguel C.M., Catalán C.A.N. (2009). The pericarp and its surface wax layer in maize kernels as resistance factors to fumonisin accumulation by *Fusarium verticillioides*. Crop Prot..

[49] Netshifhefhe N.E.I., Flett B.C., Viljoen A., Rose L.J. (2018). Inheritance and genotype by environment analyses of resistance to *Fusarium verticillioides* and fumonisin contamination in maize F1 hybrids. Euphytica.

[50] Fritsch L., Fischer R., Wambach C., Dudek M., Schillberg S., Schröper F. (2015). Next-generation sequencing is a robust strategy for the high-throughput detection of zygosity in transgenic maize. Transgenic Res..

[51] Howell S.H. (2013). Endoplasmic reticulum stress responses in plants. Annu. Rev. Plant Biol..

[52] Huttner S., Veit C., Vavra U., Schoberer J., Liebminger E., Maresch D., Grass J., Altmann F., Mach L., Strasser R. (2014). *Arabidopsis* Class I α-mannosidases MNS4 and MNS5 are involved in endoplasmic reticulum-associated degradation of misfolded glycoproteins. Plant Cell.

[53] Gidalevitz T., Stevens F., Argon Y. (2013). Orchestration of secretory protein folding by ER chaperones. Biochim. Biophys. Acta.

[54] Strasser R. (2018). Protein quality control in the endoplasmic reticulum of plants. Annu. Rev. Plant Biol..

[55] Sun X., Guo C., Ali K., Zheng Q., Wei Q., Zhu Y., Wang L., Li G., Li W., Zheng B. (2022). A Non-redundant Function of MNS5: A Class I α-1, 2 Mannosidase, in the Regulation of Endoplasmic Reticulum-Associated Degradation of Misfolded Glycoproteins. Front. Plant Sci..

[56] Hu J., Baker A., Bartel B., Linka N., Mullen R.T., Reumann S., Zolman B.K. (2012). Plant Peroxisomes: Biogenesis and Function. The Plant Cell.

[57] Terrón-Camero L., Peláez-Vico M.A., Rodriguez-Gonzalez A., del Val C., Sandalio L.M., Romero-Puertas M.C. (2022). Gene network downstream plant stress response modulated by peroxisomal H_2_O_2_. Front. Plant Sci..

[58] Beaugelin I., Chevalier A., D’Alessandro S., Ksas B., Havaux M. (2020). Endoplasmic reticulum-mediated unfolded protein response is an integral part of singlet oxygen signalling in plants. Plant J..

[59] Darino M., Jaiswal N., Darma R., Kroll E., Urban M., Xiang Y., Srivastava M., Kim H.S., Myers A., Scofield S.R. (2025). The *Fusarium graminearum* Effector Protease FgTPP1 Suppresses Immune Responses and Facilitates *Fusarium* Head Blight Disease. Mol. Plant-Microbe Interact..

[60] Jarzyniak K., Narożna D. (2024). Rapid Identification of Rhizobia Nodulating Soybean by a High-Resolution Melting Analysis. Agronomy.

[61] Tang J., Yan J., Ma X., Teng W., Wu W., Dai J., Dhillon B., Melchinger D., Li J. (2010). Dissection of the genetic basis of heterosis in an elite maize hybrid by QTL mapping in an immortalized F2 population. Theor. Appl. Genet..

[62] Sobiech A., Tomkowiak A., Jamruszka T., Kosiada T., Spychała J., Lenort M., Bocianowski J. (2025). Transcriptomic Characterization of Candidate Genes for *Fusarium* Resistance in Maize (*Zea mays* L.). Pathogens.

[63] Shapiro S.S., Wilk M.B. (1965). An analysis of variance test for normality (complete samples). Biometrika.

[64] van Eeuwijk F.A., Bink M.C.A.M., Chenu K., Chapman S.C., Malosetti M., Ribaut J.-M., van Eeuwijk F.A. (2010). The statistical analysis of multi-environment data: Modeling genotype-by-environment interaction and its genetic basis. Curr. Opin. Plant Biol..

[65] VSN International (2023). VSN International Genstat for Windows.

[66] Bocianowski J. (2024). Using NGS Technology and Association Mapping to Identify Candidate Genes Associated with Fusarium Stalk Rot Resistance. Genes.

[67] Bocianowski J., Leśniewska-Bocianowska A. (2025). Towards the Identification of Candidate Genes for Pollen Morphological Traits in *Rubus* L. Using Association Mapping. Forests.

