# Supplementary material for: DArTseq-Based, High-Throughput Identification of Novel Molecular Markers for the Detection of Fusarium Resistance in Maize
Source: Int J Mol Sci. 2025 Oct 29;26(21):10534. doi: 10.3390/ijms262110534 (PMC12609616; doi:10.3390/ijms262110534)
Supplement: Supplementary file 1 [file ijms-3940442-supplementary.pdf › ijms-3940442-supplementary.pdf]

**Table S1.** Average values of the degree of maize infection by fungi of the genus *Fusarium* for individual hybrids.

| **Hy-brid** | ***Fusarium* of cobs** | | **Hy-brid** | ***Fusarium* of cobs** | | **Hy-brid** | ***Fusarium* of cobs** | | **Hy-brid** | ***Fusarium* of cobs** | |
|---|---|---|---|---|---|---|---|---|---|---|---|
| | Mean | s.e. | | Mean | s.e. | | Mean | s.e. | | Mean | s.e. |
| G15.18 | 8.333 | 0.6667 | G17.21 | 9 | 0 | G20.02 | 8.667 | 0.3333 | G22.06 | 9 | 0 |
| G15.19 | 9 | 0 | G18.01 | 9 | 0 | G20.03 | 9 | 0 | G22.07 | 9 | 0 |
| G15.20 | 9 | 0 | G18.02 | 9 | 0 | G20.05 | 8.333 | 0.3333 | G22.08 | 8.667 | 0.3333 |
| G15.21 | 8.333 | 0.6667 | G18.03 | 8.667 | 0.3333 | G20.06 | 8.667 | 0.3333 | G22.09 | 9 | 0 |
| G16.01 | 8.667 | 0.3333 | G18.04 | 8.333 | 0.3333 | G20.07 | 9 | 0 | G22.10 | 6.667 | 0.3333 |
| G16.02 | 8.667 | 0.3333 | G18.05 | 8.667 | 0.3333 | G20.09 | 8.667 | 0.3333 | G22.11 | 8 | 0.5774 |
| G16.03 | 8.333 | 0.3333 | G18.06 | 8 | 0.5774 | G20.10 | 8.667 | 0.3333 | G22.12 | 9 | 0 |
| G16.04 | 8.667 | 0.3333 | G18.07 | 8.333 | 0.6667 | G20.11 | 9 | 0 | G22.14 | 9 | 0 |
| G16.05 | 9 | 0 | G18.08 | 8.333 | 0.6667 | G20.12 | 9 | 0 | G22.15 | 9 | 0 |
| G16.06 | 9 | 0 | G18.09 | 9 | 0 | G20.13 | 9 | 0 | G22.16 | 8.667 | 0.3333 |
| G16.07 | 9 | 0 | G18.10 | 9 | 0 | G20.14 | 8 | 0 | G22.17 | 9 | 0 |
| G16.08 | 8.667 | 0.3333 | G18.11 | 8.667 | 0.3333 | G20.15 | 9 | 0 | G22.18 | 8.667 | 0.3333 |
| G16.09 | 9 | 0 | G18.12 | 9 | 0 | G20.16 | 9 | 0 | G22.19 | 9 | 0 |
| G16.10 | 8.667 | 0.3333 | G18.13 | 8.667 | 0.3333 | G20.17 | 9 | 0 | G22.20 | 8.333 | 0.3333 |
| G16.11 | 9 | 0 | G18.14 | 9 | 0 | G20.18 | 9 | 0 | G22.21 | 9 | 0 |
| G16.12 | 8.667 | 0.3333 | G18.15 | 9 | 0 | G20.19 | 9 | 0 | G23.01 | 8.667 | 0.3333 |
| G16.13 | 8.667 | 0.3333 | G18.16 | 8.667 | 0.3333 | G20.20 | 8.667 | 0.3333 | G23.02 | 9 | 0 |
| G16.14 | 9 | 0 | G18.17 | 8.333 | 0.3333 | G20.21 | 9 | 0 | G23.03 | 8.667 | 0.3333 |
| G16.15 | 9 | 0 | G18.18 | 9 | 0 | G21.01 | 8.667 | 0.3333 | G23.04 | 9 | 0 |
| G16.16 | 8.667 | 0.3333 | G18.19 | 9 | 0 | G21.02 | 9 | 0 | G23.05 | 8.333 | 0.3333 |
| G16.17 | 8.667 | 0.3333 | G18.20 | 8 | 0 | G21.03 | 8.333 | 0.3333 | G23.06 | 8.333 | 0.3333 |
| G16.18 | 8.333 | 0.3333 | G18.21 | 9 | 0 | G21.04 | 8.667 | 0.3333 | G23.07 | 9 | 0 |
| G16.19 | 7 | 0 | G19.01 | 9 | 0 | G21.05 | 8.333 | 0.6667 | G23.08 | 9 | 0 |
| G16.20 | 6 | 0 | G19.02 | 9 | 0 | G21.06 | 9 | 0 | G23.09 | 8.667 | 0.3333 |
| G16.21 | 9 | 0 | G19.03 | 8.667 | 0.3333 | G21.07 | 9 | 0 | G23.10 | 9 | 0 |
| G17.01 | 7 | 0 | G19.04 | 9 | 0 | G21.08 | 9 | 0 | G23.11 | 8.333 | 0.3333 |
| G17.02 | 9 | 0 | G19.05 | 7 | 0.5774 | G21.09 | 9 | 0 | G23.12 | 9 | 0 |
| G17.04 | 8.667 | 0.3333 | G19.06 | 8.667 | 0.3333 | G21.10 | 9 | 0 | G23.13 | 8.333 | 0.3333 |
| G17.05 | 6 | 0 | G19.07 | 8.333 | 0.3333 | G21.11 | 9 | 0 | G23.14 | 8.667 | 0.3333 |
| G17.06 | 8.667 | 0.3333 | G19.08 | 8.667 | 0.3333 | G21.12 | 9 | 0 | G23.15 | 8 | 0 |
| G17.07 | 8.667 | 0.3333 | G19.09 | 9 | 0 | G21.13 | 7.667 | 0.6667 | G23.16 | 8.333 | 0.3333 |
| G17.08 | 8.667 | 0.3333 | G19.10 | 9 | 0 | G21.14 | 8.667 | 0.3333 | G23.17 | 9 | 0 |
| G17.09 | 8.333 | 0.3333 | G19.11 | 8.667 | 0.3333 | G21.15 | 8.667 | 0.3333 | G23.18 | 9 | 0 |
| G17.10 | 7 | 0 | G19.12 | 9 | 0 | G21.16 | 9 | 0 | G23.19 | 8 | 0 |
| G17.11 | 8.667 | 0.3333 | G19.13 | 9 | 0 | G21.17 | 9 | 0 | G23.20 | 9 | 0 |
| G17.12 | 8.667 | 0.3333 | G19.14 | 9 | 0 | G21.18 | 9 | 0 | G23.21 | 9 | 0 |
| G17.13 | 9 | 0 | G19.15 | 8.333 | 0.3333 | G21.19 | 9 | 0 | G24.01 | 8.667 | 0.3333 |
| G17.14 | 9 | 0 | G19.16 | 9 | 0 | G21.20 | 8.667 | 0.3333 | G24.02 | 9 | 0 |
| G17.15 | 8.667 | 0.3333 | G19.17 | 8.667 | 0.3333 | G21.21 | 9 | 0 | G24.03 | 9 | 0 |
| G17.16 | 8.667 | 0.3333 | G19.18 | 8.667 | 0.3333 | G22.01 | 8.667 | 0.3333 | G24.04 | 9 | 0 |
| G17.17 | 9 | 0 | G19.19 | 9 | 0 | G22.02 | 9 | 0 | G24.05 | 9 | 0 |
| G17.18 | 9 | 0 | G19.20 | 9 | 0 | G22.03 | 9 | 0 | | | |
| G17.19 | 7 | 0 | G19.21 | 8 | 0.5774 | G22.04 | 8.333 | 0.3333 | | | |
| G17.20 | 9 | 0 | G20.01 | 8.333 | 0.6667 | G22.05 | 9 | 0 | | | |
| $LSD_{0.05}$ | 0.719 | | | | | | | | | | |
